# Magnetic resonance imaging-based body composition is associated with nutritional and inflammatory status: a longitudinal study in patients with Crohn's disease

**DOI:** 10.1186/s13244-021-01121-3

**Published:** 2021-12-04

**Authors:** Ziling Zhou, Ziman Xiong, Yaqi Shen, Zhen Li, Xuemei Hu, Daoyu Hu

**Affiliations:** 1 Department of Radiology, Tongji Hospital, Tongji Medical College, Huazhong University of Science and Technology, 1095 Jiefang Avenue, Qiaokou District, Wuhan, 430030 Hubei China; 2 Biomedical Engineering Department, College of Life Sciences and Technology, Huazhong University of Science and Technology, Wuhan, China

**Keywords:** Crohn disease, Magnetic resonance enterography, Visceral adipose tissue, Drug therapy

## Abstract

**Objective:**

To evaluate the changes in magnetic resonance imaging-based body composition parameters during follow-ups in patients with Crohn's disease (CD).

**Methods:**

Between November 1, 2017, and June 30, 2021, patients diagnosed with CD, who underwent two or more magnetic resonance enterography (MRE) scans at our institution were retrospectively reviewed. The baseline and one subsequent follow-up scan for each patient were paired to form longitudinal comparisons. Skeletal muscle, visceral adipose tissue (VAT), and subcutaneous adipose tissue (SAT) indexes were calculated from tissue areas measured at the third lumbar vertebra level per scan, standardized by dividing the height^2^ and lumbar height^2^ (height_L1–L5_). We also assessed the correlation between changes in VAT to total adipose tissue ratio (VA/TA index) and CD activity scores (5-point MRE classification) using Spearman’s correlation analysis. A multivariate linear regression model was used to adjust for the follow-up duration and treatment type.

**Results:**

Overall, 49 patients (with 49 paired scans) were enrolled. VA/TA index changes were negatively correlated with changes in skeletal muscle index (SMI; *r* =  − 0.339, *p* < 0.05). The VA/TA index (52.69 ± 10.66% vs. 49.18 ± 10.80%, *p* < 0.001) and the total MRE score (8.0 ± 3.9 vs. 5.7 ± 3.4, *p* < 0.001) decreased significantly during follow-up, regardless of follow-up duration and treatment type (both *p* > 0.05). Changes in total MRE score were negatively correlated with SMI changes (*r* =  − 0.408, *p* < 0.01) but positively correlated with VA/TA index changes (*r* = 0.479, *p* < 0.01).

**Conclusion:**

An increase in SMI and a decrease in VA/TA index could reflect improved nutritional and inflammatory status.

**Supplementary Information:**

The online version contains supplementary material available at 10.1186/s13244-021-01121-3.

## Key points

1. MRI-based body composition parameters can reflect disease course in CD patients.
2. Decrease of VA/TA index correlated with improvement of disease activity during follow-up.
3. Higher SMI may indicate better nutritional status.

## Background

Crohn's disease (CD) is a chronic, inflammatory, gastrointestinal disease. According to previous studies, patients often suffer from malnutrition during the disease course [1]; thus, they face a higher risk of post-operative infections and a longer in-hospital stay [2]. Weight loss is an intuitive indication of malnutrition and has been considered one of the main features of CD; it is often accompanied by sarcopenia, which is related to inflammation, reduced physical activity, and glucocorticoid use [1, 3, 4]. Generally, CD patients were found to gain weight after anti-TNF therapy [5]. Regardless of weight gain or loss, skeletal muscle, subcutaneous fat, and visceral fat exhibit different changes during the process of weight change; however, anthropometric measurements, including abdominal circumference and body mass index (BMI)—routinely used in clinical practice—do not provide an accurate assessment of body composition changes.

Cross-sectional imaging, including computed tomography (CT) and magnetic resonance imaging (MRI), are widely used for body composition analysis [6–8]. We can obtain different body composition parameters by performing semi-automatic or manual outlining of different tissues on monolayer images at the L3 level. MRI-based quantitative analyses of muscle, visceral adipose tissue (VAT) and subcutaneous adipose tissue (SAT) have been proposed to be as reliable as CT in assessing body fat distribution and muscle content [9, 10]. Compared with CT, MR has no ionizing radiation and has a high accuracy for detecting disease activity when applied for surveillance of CD patients [11]. A link was found between MRI-based body composition parameters and inflammatory status in CD patients. For example, Büning et al. [12] found a higher proportion of VAT in women with CD than control women; Labarthe et al. [13] also found a higher proportion of VAT in patients with active CD compared to inactive patients. However, the former study group was all female, while the latter was a cross-sectional comparison of patients with different levels of activity, and few studies have assessed patients longitudinally by monitoring both changes in body composition and inflammatory status assessed by MRI during follow-up, that is, the association between changes of MRI-based body composition and changes of disease activity during treatment was still unclear.

We aimed to evaluate changes in the nutritional and inflammatory status of CD patients by comparing longitudinal clinical indicators, intestinal lesions, and MR-based body composition parameters during follow-up.

## Methods

The local review board approved this retrospective study, and the requirement of informed consent was waived.

### Patients

All patients who underwent MR enterography (MRE) scans at our institution between 1 November 2017 and 30 June 2021 were reviewed, and those with two scans and a diagnosis of CD were enrolled. The diagnosis was confirmed by an experienced inflammatory bowel disease multidisciplinary team at our institution according to the ECCO-ESGAR guidelines [14], combining information from endoscopy, biopsy, laboratory tests, imaging findings, and history review. Patients were further excluded if: (1) they underwent bowel resection between their first and subsequent scans; (2) clinical data on treatment information or laboratory information reflecting nutritional status between two scans was unavailable.

After identifying patients who met the above criteria, information (sex, age at inclusion, family history, smoking history) at the time of the first MRE per patient was recorded from the electronic medical record system. Clinical data (age, height, weight, BMI, specific CD-related treatment, erythrocyte sedimentation rate (ESR), C-reactive protein (CRP), albumin (Alb), and haematocrit (Hct) levels) and the Montreal classification [15] were recorded within seven days of each MRE scan. BMI was obtained according to the formula BMI = body weight (kg)/height^2^ (m^2^) and was categorized as normal (18.5–24.9), underweight (< 18.5), and overweight (25–30) [16]. We paired each patient's initial scan with one subsequent follow-up scan to form a longitudinal comparison group.

### MRE examinations

All examinations are performed on one of four 3.0 T MR scanners (GE Discovery 750HD, GE Healthcare; Siemens MAGNETOM Skyra (2 sets), Siemens AG; uMR 780, United Imaging Healthcare). All patients underwent pre-scan preparation as recommended by the European Society of Gastrointestinal and Abdominal Radiology (ESGAR) and European Society of Pediatric Radiology (ESPR) [17]; that is, fasting for 4–6 h before the scan and drinking 1000–1500 mL of 2.5% aqueous mannitol solution regularly within 30–45 min before the scan. To minimize bowel peristalsis, 10 mg anisodamine was orally administered 30 min before the examination to patients without contraindications, including glaucoma and prostatic hypertrophy. Additionally, routine MRE sequences were consistent with those suggested by ESGAR/ESPR, as shown in the Additional file 1: Table S1.

### Images analysis

Digital imaging and communications in medicine images of the MRE sequence were extracted per patient for quantification of body composition parameters and assessment of inflammatory involvement of the intestine.

#### Quantification of body composition parameters

**Fig. 1:**
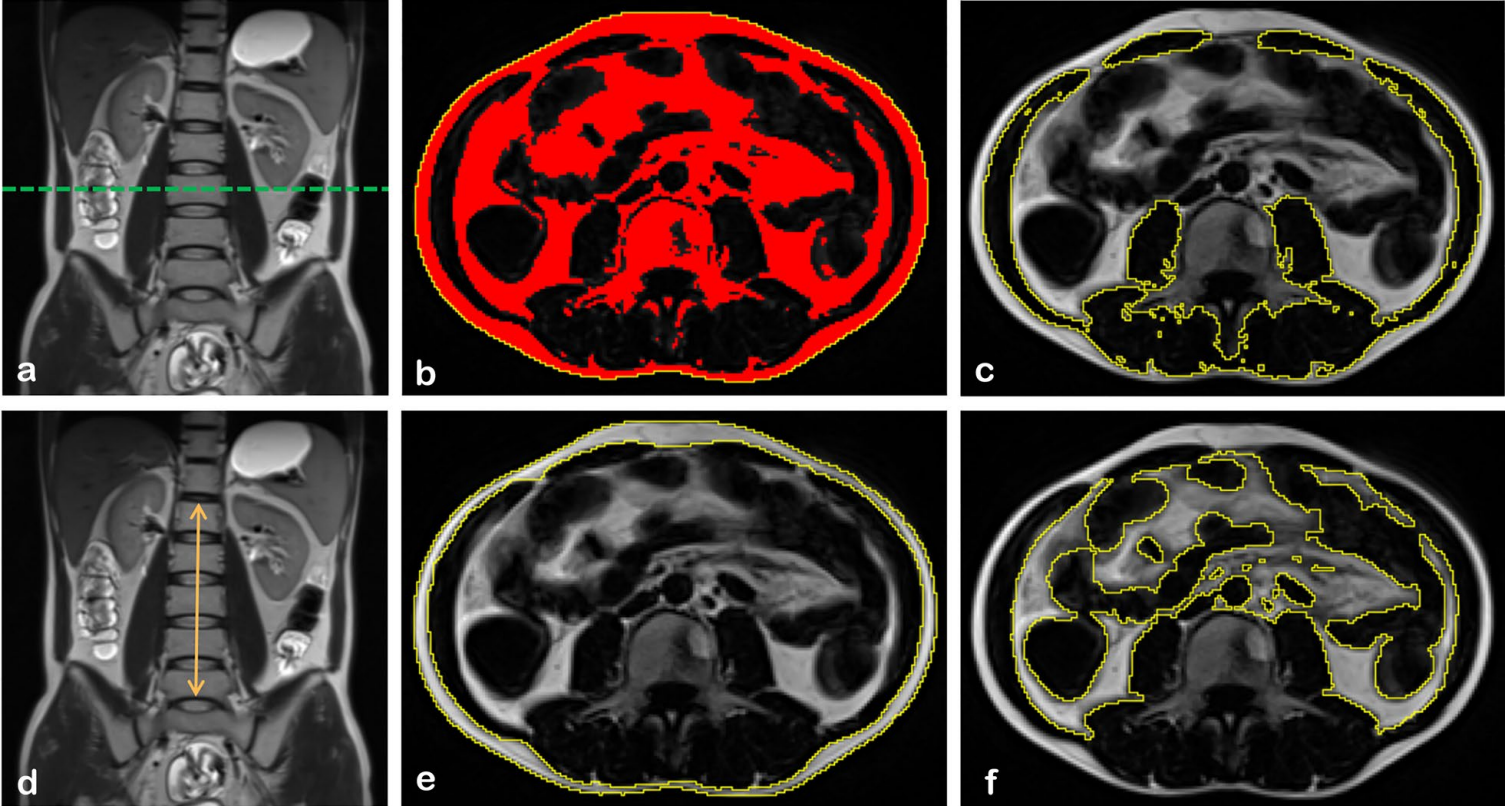
Extraction diagram of body composition parameters. **a** Selection of axial L3 level T1WI slice using coronal T2WI as a reference; **b** mask generated by ImageJ; **c** region of interest of muscle segmented manually based on a generated mask; **d** example showing the method to measure the lumbar height; **e** region of interest of SAT segmented manually based on a generated mask; **f** region of interest of VAT segmented manually based on the generated mask. SAT: subcutaneous adipose tissue, VAT: visceral adipose tissue

One research assistant extracted a single slice at the mid-level of the third lumbar vertebra (L3) from the axial T1-weighted images for further evaluation. Segmentation and measurement of skeletal muscle, SAT, and VAT were performed by a semi-automated method [18] using ImageJ (National Institutes of Health, https://imagej.nih.gov/ij/index.html), as shown in Fig. 1. Thereby, we obtained cross-sectional areas (cm^2^) of skeletal muscle, SAT, and VAT. Additionally, we measured the height between the upper edge of the first and lower edge of the fifth lumbar vertebrae in each scan (height_L1–L5_, cm), which is illustrated in Fig. 1. The quantification processes were separately performed by two radiologists blinded to the patient's medical information; the average of the two measurements was used for subsequent analysis. Additionally, the skeletal muscle, SAT, and VAT areas were normalized by both height square (m^2^) and height_L1–L5_ square (cm^2^) to obtain the skeletal muscle index (SMI and SMI_spinal_), subcutaneous adipose index (SAI and SAI_spinal_), and visceral adipose index (VAI and VAI_spinal_), respectively. Sarcopenia was defined as: SMI < 49.9 cm^2^/m^2^ for men; and SMI < 28.7 cm^2^/m^2^ for women, according to previously defined cut-off value [19]. We calculated the percentage of VAT to total adipose tissue (VA/TA index) in a single layer based on the formula VAT area/(SAT area + VAT area) × 100%.

#### Qualitative assessment of the intestine

We evaluated the intestinal lesions in each scan, referring to the 5-Point MR enterocolonography classification [20]. The procedure consists of visual scoring of the jejunum, distal ileum, terminal ileum, ascending colon, transverse colon, descending colon, sigmoid colon, and rectum, where there is: 0: normal or no lesions; 1: inactive or quiescent CD; 2: mild active inflammation; 3: moderate active inflammation; 4: severe active inflammation. Two radiologists evaluated each scan, and any inconsistencies between them were reviewed by another senior radiologist, and a final decision was made accordingly. The MRE scores for the small bowel (jejunum, distal ileum, and terminal ileum) and colorectum (colon and rectum) were calculated separately and then added together to obtain the total MRE score for each scan. According to the MRE score criteria proposed by Kitazume et al., we defined intestinal improvement as: a reduction in total MRE score ≥ 1, and no intestinal segment score increased at follow-up.

### Statistical analysis

Statistics were conducted using SPSS 26.0 (IBM Co.) and R software version 3.6.0. Quantitative and categorical variables were described as mean ± standard deviation (SD) and frequencies (%), respectively. A paired Student's t-test or a Wilcoxon signed-rank test was performed as appropriate; categorical variables were compared through Chi-squared or Fisher's exact tests. Additionally, we calculated the changes in clinical indicators, body composition parameters, and MRE scores for each longitudinal comparison group at follow-up versus baseline to obtain change values (ΔBMI, ΔCRP, ΔESR, ΔAlb, ΔHct, ΔSMI, ΔSAI, ΔVAI, ΔVA/TA index, and ΔMRE score). The specific calculation is as follows: Δ(*i*) = (*i*)Value_follow-up_ – (*i*)Value_baseline_. Correlations between clinical and MR changes, height and height_L1-L5_, and standardized indices (SMI and SMI_spinal_, SAI and SAI_spinal_, and VAI and VAI_spinal_) were evaluated using Spearman/Pearson correlation analysis (0.3 ≤|*r*|< 0.7, moderate; |*r*|> 0.7, high). Correlation between body composition parameters changes and total MRE score changes were further adjusted with age, sex, follow-up duration and treatment type using multivariate linear regression analysis. Follow-up duration was defined as the interval between two MRE scans per patient. Treatment type was categorized as biologic therapy and non-biologic therapy (other therapies without induction of biological agent). Intraclass correlation coefficient (ICC) or weighted kappa coefficient was used to assess the reliability of body composition area measurements and bowel assessment results between two radiologists; two-tailed *p*-values < 0.05 were considered significant.

## Results

**Fig. 2:**
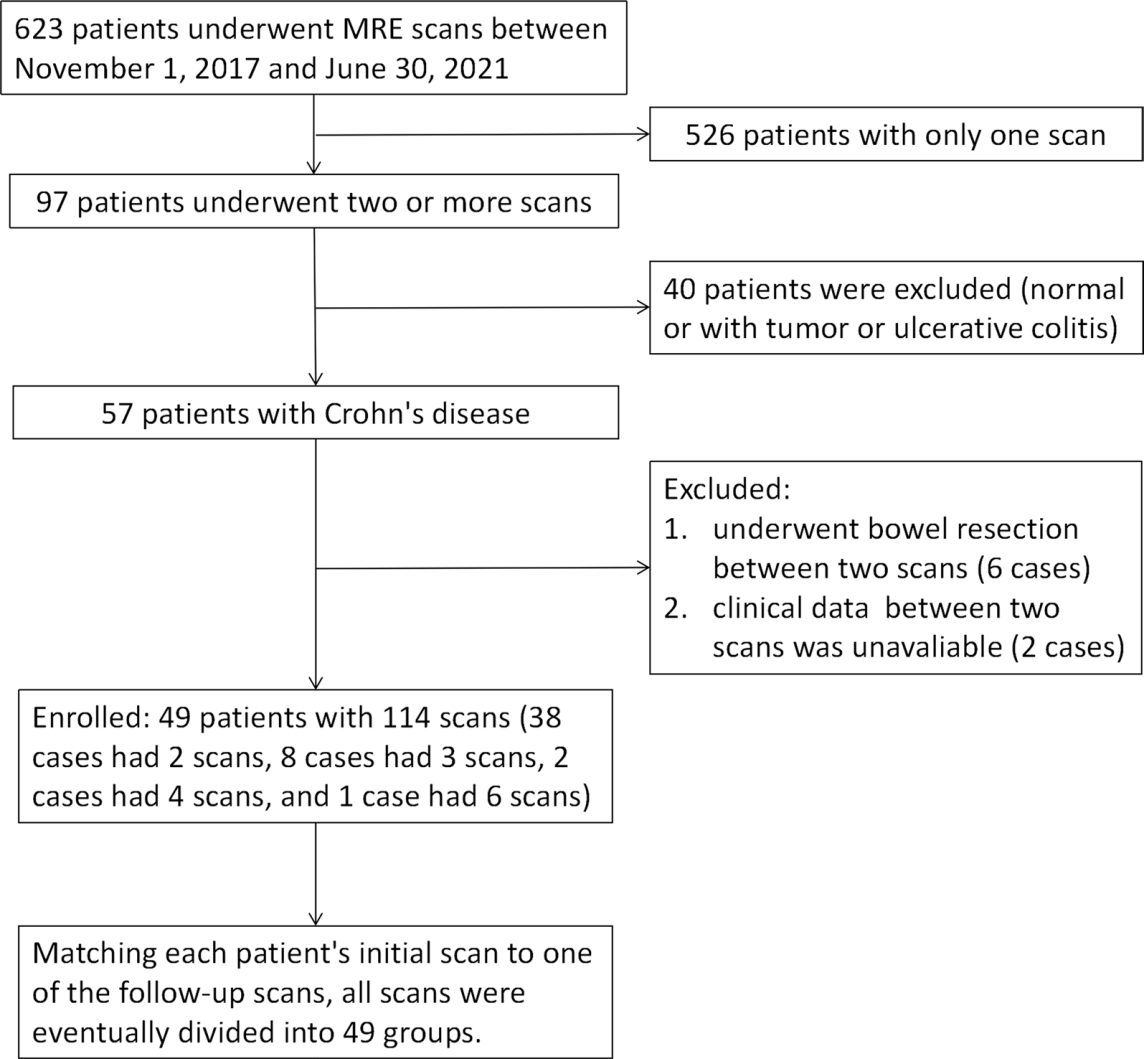
Inclusion flowchart for Crohn's disease cases

There were 49 patients enrolled in this study (Fig. 2). Two MRE scans per patient comprised the longitudinal comparison groups and were used to extract body composition parameters further.

### Clinical characteristics

The demographic characteristics of all CD patients are shown in Table 1. Baseline and follow-up clinical information for all patients are shown in Table 2, where baseline information refers to the data obtained when the patients had their first MRE scan at our facility. Patients were predominantly young males, and none indicated a family history of CD. Thirty-eight (77.6%) patients were treated with biological agents (predominantly infliximab, 29/38 cases), seven with enteral nutrition, three with immunosuppressants (azathioprine), and corticosteroids, and one with 5-ASA. During the follow-up period, the patient's disease site and behaviour did not change significantly. Various laboratory parameters statistically changed, including a significant decrease in CRP and ESR and a significant increase in Alb and Hct (*p* < 0.001). BMI values of all patients increased (18.27 ± 3.04 kg/m^2^ vs. 19.55 ± 2.66 kg/m^2^; *p* < 0.001), and there was also a significant increase in the proportion of patients within the normal BMI range (36.7% vs. 57.1%).

**Table 1:** Clinical features of all Crohn's disease patients

Characteristics	Baseline (*n* = 49)
Age at inclusion, years (mean ± SD)	25.82 ± 9.90
Gender (male: female)	3.9:1
Active smokers, n (%)	10 (20.4)
Scanning interval, months (median, IQR)	4.5 (3.5, 7.5)
Treatment, *n* (%)	
Biological agents	38 (77.6)
Infliximab	29
Adalimumab	4
Vedolizumab	3
Ustekinumab	2
Enteral nutrition	7 (14.3)
Immunosuppressant + corticosteroid	3 (6.1)
5-ASA	1 (2.0)

*SD* Standard deviation, *IQR* interquartile range, *5-ASA* 5-aminosalicylic acid

**Table 2:** Clinical data of all Crohn's disease patients at initial and follow-up scan

Characteristics	Baseline (*n* = 49)	Follow-up (*n* = 49)	*p*-value
Montreal classification			
Age at diagnosis, n (%)			
A1	11 (22.4)		
A2	34 (69.4)		
A3	4 (8.2)		
Location, n (%)			0.93
L1	17 (34.7)	15 (30.6)	
L2	1 (2.0)	2 (4.1)	
L3	30 (61.2)	31 (63.3)	
L3 + 4	1 (2.0)	1 (2.0)	
Behaviour, n (%)			0.93
B1	31 (63.3)	28 (57.1)	
B2	11 (22.4)	13 (26.5)	
B3	5 (10.2)	6 (12.2)	
B2 + 3	2 (4.1)	2 (4.1)	
Perianal disease, *n* (%)	28 (57.1)	29 (59.2)	1.00
BMI, kg/m^2^ (mean ± SD)	18.27 ± 3.04	19.55 ± 2.66	**< 0.001**
BMI classification, *n* (%)			**0.047**
Normal	18 (36.7)	28 (57.1)	
Underweight	29 (59.2)	21 (42.9)	
Overweight	2 (4.1)	0	
Laboratory findings (mean ± SD)			
CRP, mg/L (*n* = 46)	35.34 ± 43.39	11.71 ± 20.98	**< 0.001**
ESR, mm/h (*n* = 48)	23.85 ± 17.97	11.04 ± 13.51	**< 0.001**
Alb, g/L (*n* = 46)	38.43 ± 5.85	42.19 ± 5.42	**< 0.001**
HCT, % (*n* = 47)	34.70 ± 6.15	38.71 ± 5.71	**< 0.001**

*p* value of variables with great statistical difference between baseline and follow-up is marked in bold

*SD* Standard deviation, *BMI* body mass index, *CRP* C-reactive protein, *ESR* erythrocyte sedimentation rate, *Alb* albumin, *Hct* haematocrit

### Body composition and intestinal lesion assessment

**Fig. 3:**
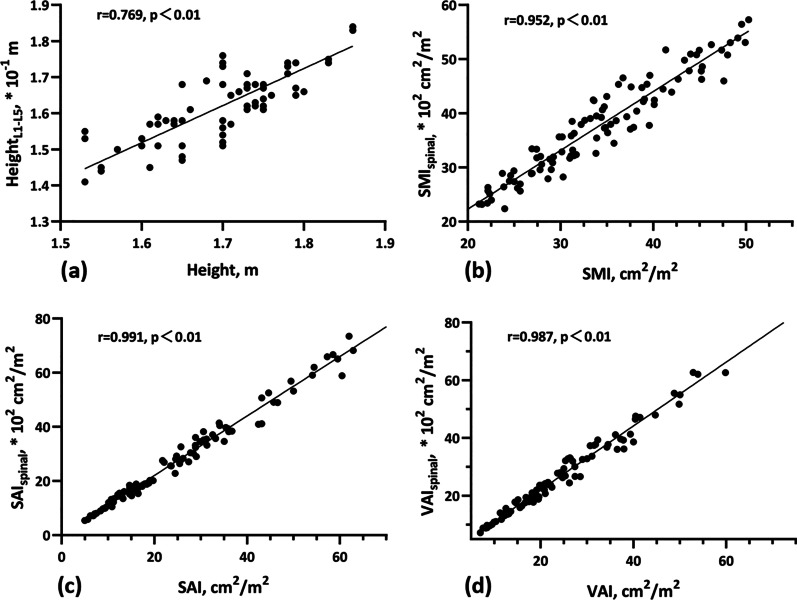
Scatter plots showing the significant positive correlations between height and the height of the 1st to 5th lumbar vertebrae (**a**), as well as between the standardized indices obtained via two different methods (**b–d**)

By analysing patients' heights and lumbar spine heights in all 98 scans, we found a high correlation between height and height_L1-L5_ (Fig. 3a, *r* = 0.769, *p* < 0.01). Additionally, high correlations were observed between SMI and SMI_spinal_, SAI and SAI_spinal_, and VAI and VAI_spinal_ (Fig. 3b–d, *r* = 0.952, 0.991 and 0.987, respectively; *p* < 0.01).

The ICCs for inter-rater reliability in quantitative measurements of skeletal muscle, SAT, and VAT area were 0.982, 0.999, and 0.999, respectively (*p* < 0.001). For the qualitative assessment of the intestine, the weighted kappa coefficients for each segment were shown in Additional file 1: Table S2, and the total MRE scores obtained were also well reproducible (ICC = 0.716, *p* < 0.001).

### Changes in body composition and MRE scores during follow-up

**Fig. 4:**
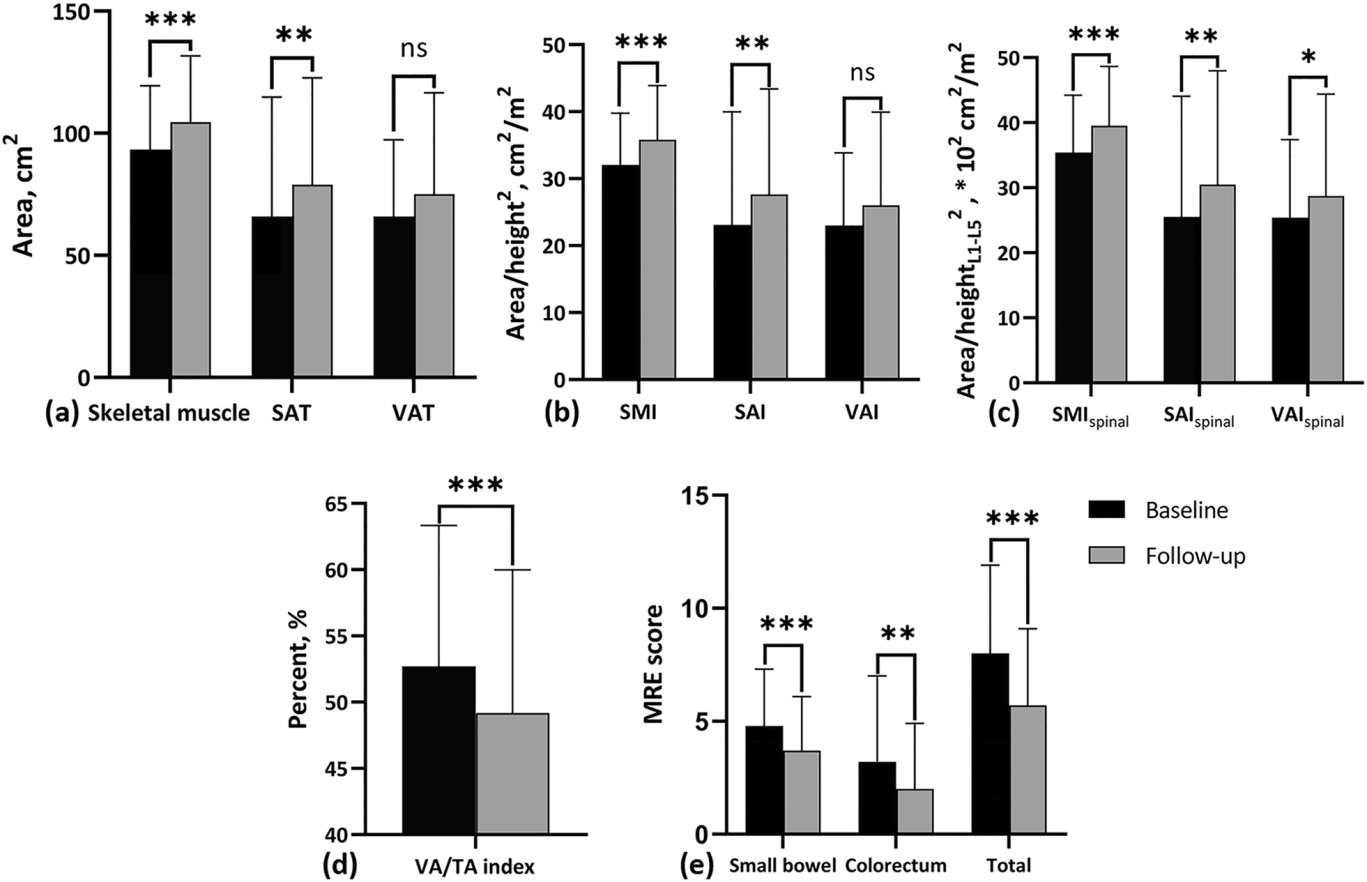
Bar graphs of body composition parameters and MRE scores for all cases. Black bars represent baseline data, and grey bars represent follow-up data. **a** Area of skeletal muscle, SAT, and VAT at baseline and follow up; **b** Value of SMI, SAI, and VAI at baseline and follow up; **c** Value of SMIspinal, SAIspinal, and VAIspinal at baseline and follow up; **d** Value of VA/TA index at baseline and follow up; **e** MRE scores of small bowel and colorectum at baseline and follow up. ***: *p* < 0.001, **: *p* < 0.01, *: *p* < 0.05, ns: *p* > 0.05 (not significant); SAT: subcutaneous adipose tissue, VAT: visceral adipose tissue, SMI, skeletal muscle index; SAI, subcutaneous adipose index; VAI, visceral adipose index; SMI_spinal_, skeletal muscle area normalized by lumbar spinal height^2^; SAI_spinal_, subcutaneous adipose tissue area divided by lumbar spinal height^2^; VAI_spinal_, visceral adipose tissue area divided by lumbar spinal height^2^; ns, no significant difference; VA/TA index, VAT to total adipose tissue ratio

**Fig. 5:**
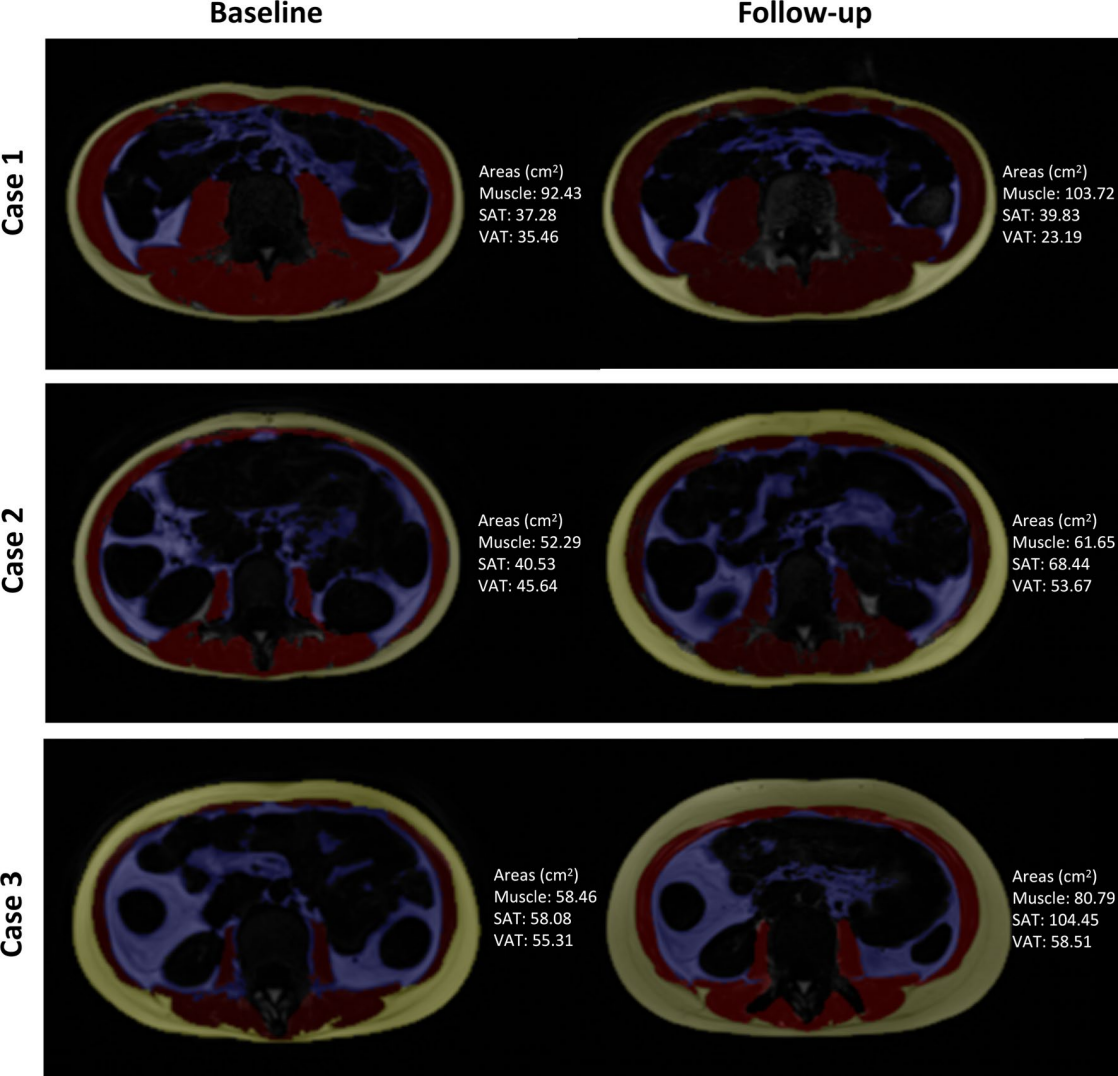
Changes in body composition area during follow-up in three Crohn's disease cases. The area of each component in these images has been labeled, and the changes in VA/TA index of case 1–3 is − 11.95%, − 9.01% and − 12.88%, respectively. SAT: subcutaneous adipose tissue, VAT: visceral adipose tissue, VA/TA index: VAT to total adipose tissue ratio

As shown in Fig. 4a–c, area of skeletal muscle (93.26 ± 26.15 cm^2^ vs. 104.50 ± 27.24 cm^2^) and SAT (65.88 ± 48.87 cm^2^ vs. 78.93 ± 43.70 cm^2^) increased significantly during follow-up (*p* < 0.001 and *p* < 0.01, respectively). Representative images showing the body composition area changes in three patients are illustrated in Fig. 5. Accordingly, indexes including SMI (32.08 ± 7.71 cm^2^/m^2^ vs. 35.83 ± 8.05 cm^2^/m^2^), SMI_spinal_ (35.41 ± 8.81 * 10^2^ cm^2^/m^2^ vs. 39.51 ± 9.14 * 10^2^ cm^2^/m^2^), SAI (23.10 ± 16.87 cm^2^/m^2^ vs. 27.65 ± 15.75 cm^2^/m^2^), and SAI_spinal_ (25.50 ± 18.58 * 10^2^ cm^2^/m^2^ vs. 30.46 ± 17.51 * 10^2^ cm^2^/m^2^) showed the same increasing trend during follow-up (*p* < 0.001 for SMI and SMI_spinal_, and *p* < 0.01 for SAI and SAI_spinal_, respectively). No significant changes were observed in VAT area (65.88 ± 31.37 cm^2^ vs. 75.10 ± 41.49 cm^2^) and VAI (23.01 ± 10.85 cm^2^/m^2^ vs. 26.00 ± 13.92 cm^2^/m^2^) (*p* > 0.05). VAI_spinal_ (25.39 ± 12.00 * 10^2^ cm^2^/m^2^ vs. 28.72 ± 15.66 * 10^2^ cm^2^/m^2^) showed a slightly increase during follow-up (*p* = 0.04). On the contrary, VA/TA index decreased significantly during the same process (52.69 ± 10.66% vs. 49.18 ± 10.80%, *p* < 0.001) (Fig. 4d). Sarcopenia presented in all patients at the time of the first MRE scan and presented in 37 males and 7 females after follow-up (*p* = 0.77). Additionally, the small bowel, colorectum and total MRE scores all decreased significantly during the follow-up period (Fig. 4e).

### Correlation among clinical indicators changes, MRE score changes, and body composition changes during follow-up

**Fig. 6:**
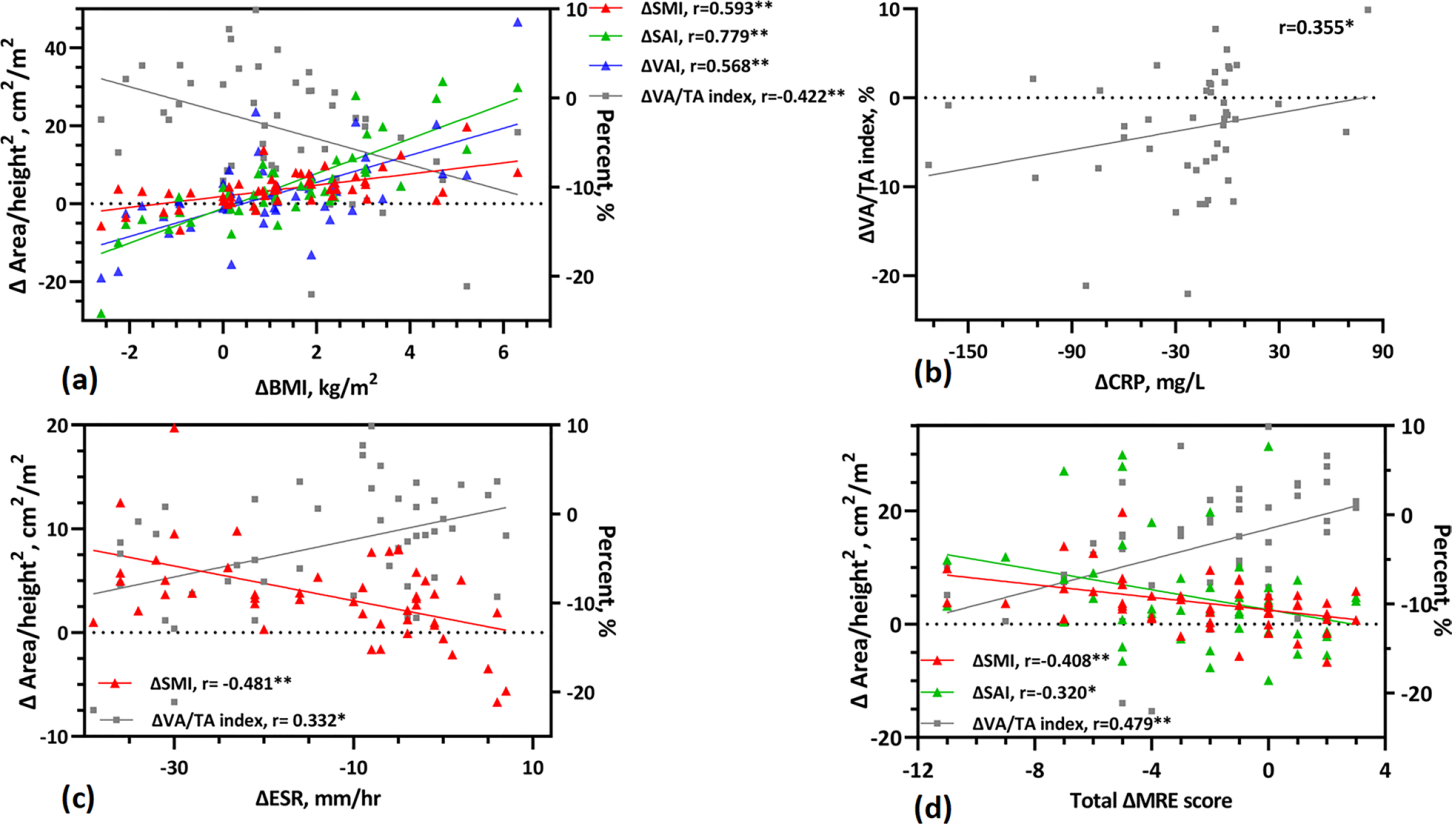
Scatter plots showing the correlation between changes in clinical indicators, MRE score, and body composition parameters during follow-up. **a** Correlation between changes of body composition parameters (ΔSMI, ΔSAI, ΔVAI, and ΔVA/TA) and BMI; **b** Correlation between ΔVA/TA and ΔCRP; **c** Correlation between changes of body composition parameters (ΔSMI and ΔVA/TA) and ESR; **d** Correlation between changes of body composition parameters (ΔSMI, ΔSAI, and ΔVA/TA) and total MRE score. **: *p* < 0.01, *: *p* < 0.05; BMI, body mass index; CRP, C-reactive protein; ESR, Erythrocyte sedimentation rate; SMI, skeletal muscle index; SAI, subcutaneous adipose index; VAI, visceral adipose index, VA/TA index: VAT to total adipose tissue ratio; Δ(*i*) =(*i*)Value_follow-up_ − (*i*)Value_baseline_, *i*= BMI, SMI, SAI, VAI, VA/TA, CRP, ESR, MRE score

The correlation coefficients for changes in clinical indicators, MRE score, and body composition parameters are shown in Additional file 1: Table S3. We found that ΔBMI was moderately to highly positively correlated with ΔSMI, ΔSAI, and ΔVAI (*r* = 0.593, 0.779, and 0.568, respectively; *p* < 0.01), and moderately negatively correlated with the ΔVA/TA index (*r* =  − 0.422; *p* < 0.01) (Fig. 6a). The correlation between ΔCRP and body composition parameters was only reflected in the ΔVA/TA index (*r* = 0.355, *p* < 0.05) (Fig. 6b). ΔESR was moderately negatively correlated with ΔSMI (*r* =  − 0.481, *p* < 0.01) but positively correlated with ΔVA/TA index (*r* = 0.332, *p* < 0.05) (Fig. 6c). The Δtotal MRE score showed a negative correlation with both ΔSMI and ΔSAI (*r* =  − 0.408, *p* < 0.01; − 0.320, *p* < 0.05) and a positive correlation with the ΔVA/TA index (*r* = 0.479, *p* < 0.01) (Fig. 6d). The correlation between ΔVAI and Δtotal MRE score was not observed (*r* =  − 0.020, *p* > 0.05). Additionally, results showed that ΔSMI was positively correlated with both ΔAlb and ΔHct (*r* = 0.450 and 0.422, respectively *p* < 0.01) and negatively correlated with ΔVA/TA index (*r* =  − 0.339, *p* < 0.05). Furthermore, we found that the differences in SMI, SAI, and VAI during follow-up were mainly in patients using biological agents, while the differences in VA/TA index were significant for all therapy methods. Additionally, the patients' intestinal improvement was not affected by the treatment modality (Table 3). When adjusted by variates, including sex, age, follow-up duration, and treatment type, ΔVA/TA index was still strongly associated with the Δtotal MRE score (Table 4).

**Table 3:** Effect of therapy on body composition parameters and intestinal improvement

	Biological agents (*n* = 38)	Non-biological agents (*n* = 11)	*P*1	*P*2	*P*3
ΔSMI, cm^2^/m^2^	4.38 ± 4.61	1.62 ± 4.46	**< 0.001**	0.26	**0.0499**
ΔSAI, cm^2^/m^2^	4.51 ± 8.07	4.68 ± 17.13	**< 0.001**	0.39	0.93
ΔVAI, cm^2^/m^2^	3.75 ± 10.47	0.37 ± 11.22	**0.017**	0.92	0.61
ΔVA/TA index, %	− 3.22 ± 7.23	− 4.51 ± 4.63	**0.009**	**0.009**	0.58
Intestinal improvement, *n*	24	6	–	–	0.73

*p* values below 0.05 are marked in bold

*SMI* Skeletal muscle index, *SAI* subcutaneous adipose tissue index, *VAI* visceral adipose tissue index, *VA/TA* area of visceral fat/area of subcutaneous fat; *P*1 intra-group differences between baseline and follow-up values in patients group with biologic therapy type; *P*2 intra-group differences between baseline and follow-up values in patients group with non-biologic therapy type; *P*3 inter-group differences evaluated using the independent samples *t*-test or Mann–Whitney *U* test, and the *χ*^2^ test

**Table 4:** Correlation between body composition parameters changes and Δtotal MRE scores with adjustment of multi-variables

Variables	ΔVA/TA			ΔSMI			ΔSAI		
	Coefficient	SD	*p* value	Coefficient	SD	*p* value	Coefficient	SD	*p* value
Intercept	− 7.69	3.13	0.017	2.10	2.31	0.369	3.19	5.06	0.532
Biologic therapy	2.15	2.06	0.302	1.90	1.52	0.219	− 1.49	3.33	0.656
Follow-up duration	0.16	0.16	0.308	0.02	0.12	0.868	0.68	0.25	**0.010**
Age	0.15	0.09	0.092	− 0.03	0.06	0.632	− 0.17	0.14	0.226
Sex (female)	− 2.48	2.10	0.245	− 1.31	1.55	0.403	5.46	3.41	0.117
Δtotal MRE scores	0.84	0.25	**0.001**	− 0.50	0.19	**0.010**	− 0.67	0.41	0.109

*p* values below 0.05 are marked in bold

*MRE* Magnetic resonance enterography, *SMI* skeletal muscle index, *SAI* subcutaneous adipose tissue index, *VA/TA* area of visceral fat/area of subcutaneous fat

## Discussion

This retrospective, longitudinal study explored changes in clinical indicators, disease activity, and body composition parameters in CD patients during follow-up. In the present study, the change in VAT percentage (VA/TA index) and SMI during treatment in CD patients can be used as feasible indicators to monitor overall nutritional and inflammatory status, since ΔVA/TA index was correlated well with changes in CRP, ESR and MRE activity score, while ΔSMI correlated linearly with changes in BMI, Alb, Hct, and MRE activity score during follow-up.

After treatment, a significant increase in skeletal muscle, SAT, and VAT area was found, while an unexpected decrease in the relative visceral fat content (VA/TA index) was observed. The changes in skeletal muscle, SAT, and VAT during treatment were positively correlated with the evolution of BMI, whereas the VA/TA index was negatively correlated. Drug therapy-induced weight gain in CD patients has been reported [5]. A prospective study found that anti-TNF therapy caused a rapid and uniform gain in abdominal fat; i.e., the increase occurred uniformly in both SAT and VAT [21]. This is somewhat similar to findings in the present study; however, they did not observe a link between these indicators and trends in BMI nor conduct further analysis targeting the relative VAT content.

Adipose tissue has been described as an endocrine organ that profoundly impacts metabolism [22]. In humans, SAT and VAT have been shown to have significantly different gene expression patterns [23, 24]. VAT inflammation has long been recognized as important in the pathogenesis of CD; its encapsulation of diseased intestinal segments to form the so-called “creeping fat” is an important source of pro-inflammatory cytokines (e.g., IL-6 and TNF-α) [25–27]. Contrary to the negative effects of abnormal VAT accumulation, several studies have confirmed SAT to benefit metabolism [28–30]. The present study showed that the ΔVA/TA index was positively correlated with the trends in CRP, ESR, and total MRE score (*r* = 0.355, *p* < 0.05; *r* = 0.332, *p* < 0.05; *r* = 0.479, *p* < 0.01), while ΔSAI was negatively correlated with Δtotal MRE score (*r* =  − 0.320, *p* < 0.05). Labarthe et al. [13] also observed that active/severe CD patients had a higher VA/TA index and lower SAI than inactive patients, similar to the present results. Still, we focus more on analysing changes in disease over treatment, which is more applicable to monitoring during follow-up. Therefore, changes in the two fat parameters could reflect changes in the inflammatory state.

Although data indicates that the prevalence of overweight and obesity is increasing in the CD population and that increased VAT caused by obesity promotes disease progression [31], in patients with a low BMI, hypertrophic mesenteric adipose tissue is observed early in the onset of the disease [32]. Moreover, even though most CD patients exhibited a normal or even low BMI, they still had a higher percentage of VAT when compared with healthy subjects [33]. In the present study, twenty-nine patients (59.2%) had low baseline BMIs. After treatment, their overall BMI had improved, while a significant reduction in VAT percentage occurred at the same time. Therefore, we believe that BMI may be misleading and does not provide a good indication of changes in abnormal VAT for accurate determination of disease progression status.

Monitoring the nutritional status of CD patients during treatment is important for their quality of life [34]. Skeletal muscle content and density are indexes to assess patients' nutritional condition in previous studies, and sarcopenia is often used to describe a combined loss of muscle function and mass. Wiese et al. [35] presented that infliximab reversed poor nutritional status in CD, while Subramaniam et al. [36] showed increased muscle mass in patients treated with anti-TNF. All patients in this study suffered sarcopenia at their inclusion. After subsequent treatment, their nutritional status showed an overall improvement, as evidenced by an increase in SMI and a decrease in patients who suffered sarcopenia. A consistent finding is that the increase in SMI was paralleled by improvements in BMI and laboratory nutritional index like Alb and Hct, as ΔSMI was positively correlated with trends in Alb and Hct (*r* = 0.450 and 0.422, respectively, *p* < 0.01). Additionally, the increase of SMI and decrease of VA/TA index were strongly associated with improvement in disease activity reflected by the decrease of total MRE score after adjustment of multiple variables. An explanation for this is that CD patients often have nutrient absorption issues, mainly reflected in reduced fat and protein content, leading to amino acid deficiency and hypoproteinaemia [37]. Hypoproteinaemia occurs not only due to decreased synthesis but also increased catabolism during inflammation [3]. Therefore, changes in the nutritional status and disease activity of CD patients during treatment can be well detected by body composition parameters, including SMI and VA/TA index.

In this study, a method for area normalization using height_L1–L5_ instead of height was proposed. We found a strong correlation between the standardized indices of skeletal muscle, SAT, and VAT obtained by the two routes (*r* = 0.952, 0.991, and 0.987, respectively; *p* < 0.01). Height_L1-L5_ is predominant in conditions when accurate standing height data was unavailable, like emergency admission. If height can be replaced by height_L1–L5_ measured directly on the images, patients excluded due to lack of height data can be decreased and thus increase the statistical power of similar retrospective studies. Further large-scale prospective research was needed to facilitate the application of height_L1–L5_ in body composition analysis.

This study has some limitations. First, due to its retrospective design, some clinical information like endoscopic data was not available at the time of each MRE scan. Correspondingly, we evaluated the disease activity of each patient by scoring the intestinal lesions in each MRE image. Information was also incomplete for calculating the Crohn's disease activity index in most CD patients at their admission. Further study is needed for verifying the significant changes of SMI and VA/TA with inflammatory status reflected by endoscopic data or Crohn's disease activity index. Second, selection bias may exist due to the relatively small sample size and single centre design. Finally, although the current semi-automated segmentation method can obtain accurate body composition parameters, it is still a time-consuming process. To promote clinical practicability, the same precise automated segmentation methods should be developed in future works.

In conclusion, body composition analysis based on MRI is reproducible; the information obtained, especially the SMI and VA/TA index, can be used to monitor the nutritional and inflammatory status of CD patients during follow-up.

## Supplementary Information

**Additional file 1.** **Table S1.** MRE protocol. **Table S2.** Kappa coefficients for each intestinal segment in the MRE assessment. **Table S3.** Correlation between changes in body composition and clinical indicators during treatment (Spearman coefficient or Pearson coefficient).

## Data Availability

The datasets used or analyzed during the current study are available from the corresponding author on reasonable request.

## Abbreviations

Alb: Albumin
BMI: Body mass index
CD: Crohn's disease
CRP: C-reactive protein
ESR: Erythrocyte sedimentation rate
Hct: Haematocrit
MRE: Magnetic resonance enterography
SAI: Subcutaneous adipose index
SAT: Subcutaneous adipose tissue
SMI: Skeletal muscle index
VAI: Visceral adipose index
VAT: Visceral adipose tissue

## References

[1] Balestrieri P, Ribolsi M, Guarino MPL, Emerenziani S, Altomare A, Cicala M (2020). Nutritional aspects in inflammatory bowel diseases. Nutrients.

[2] Takaoka A, Sasaki M, Nakanishi N (2017). Nutritional screening and clinical outcome in hospitalized patients with Crohn's disease. Ann Nutr Metab.

[3] Stokes MA (1992). Crohn's disease and nutrition. Br J Surg.

[4] Grillot J, D'Engremont C, Parmentier AL (2020). Sarcopenia and visceral obesity assessed by computed tomography are associated with adverse outcomes in patients with Crohn's disease. Clin Nutr.

[5] Christian KE, Russman KM, Rajan DP, Barr EA, Cross RK (2020). Gender differences and other factors associated with weight gain following initiation of infliximab: a post hoc analysis of clinical trials. Inflamm Bowel Dis.

[6] Grossberg AJ, Chamchod S, Fuller CD (2016). Association of body composition with survival and locoregional control of radiotherapy-treated head and neck squamous cell carcinoma. JAMA Oncol.

[7] Weston AD, Korfiatis P, Kline TL (2019). Automated abdominal segmentation of CT scans for body composition analysis using deep learning. Radiology.

[8] Huber FA, Chaitanya K, Gross N (2021). Whole-body composition profiling using a deep learning algorithm: influence of different acquisition parameters on algorithm performance and robustness. Invest Radiol.

[9] Seidell JC, Bakker CJ, van der Kooy K (1990). Imaging techniques for measuring adipose-tissue distribution—a comparison between computed tomography and 1.5-T magnetic resonance. Am J Clin Nutr.

[10] Baum T, Cordes C, Dieckmeyer M (2016). MR-based assessment of body fat distribution and characteristics. Eur J Radiol.

[11] Rimola J, Rodriguez S, García-Bosch O (2009). Magnetic resonance for assessment of disease activity and severity in ileocolonic Crohn's disease. Gut.

[12] Büning C, von Kraft C, Hermsdorf M (2015). Visceral adipose tissue in patients with Crohn's disease correlates with disease activity, inflammatory markers, and outcome. Inflamm Bowel Dis.

[13] Labarthe G, Dolores M, Verdalle-Cazes M (2020). Magnetic resonance imaging assessment of body composition parameters in Crohn's disease. Dig Liver Dis.

[14] Maaser C, Sturm A, Vavricka SR (2019). ECCO-ESGAR guideline for diagnostic assessment in IBD Part 1: initial diagnosis, monitoring of known IBD, detection of complications. J Crohns Colitis.

[15] Satsangi J, Silverberg MS, Vermeire S, Colombel JF (2006). The Montreal classification of inflammatory bowel disease: controversies, consensus, and implications. Gut.

[16] WHO Expert Consultation (2004). Appropriate body-mass index for Asian populations and its implications for policy and intervention strategies. Lancet.

[17] Taylor SA, Avni F, Cronin CG (2017). The first joint ESGAR/ESPR consensus statement on the technical performance of cross-sectional small bowel and colonic imaging. Eur Radiol.

[18] Gomez-Perez S, McKeever L, Sheean P (2020). Tutorial: a step-by-step guide (version 2.0) for measuring abdominal circumference and skeletal muscle from a single cross-sectional computed-tomography image using the national institutes of health image. J Parenter Enteral Nut.

[19] Zhang TH, Ding C, Xie TB (2017). Skeletal muscle depletion correlates with disease activity in ulcerative colitis and is reversed after colectomy. Clin Nutr.

[20] Kitazume Y, Fujioka T, Takenaka K (2019). Crohn disease: a 5-point MR enterocolonography classification using enteroscopic findings. AJR Am J Roentgenol.

[21] Parmentier-Decrucq E, Duhamel A, Ernst O (2009). Effects of infliximab therapy on abdominal fat and metabolic profile in patients with Crohn's disease. Inflamm Bowel Dis.

[22] Pantanetti P, Garrapa GGM, Mantero F, Boscaro M, Faloia E, Venarucci D (2004). Adipose tissue as an endocrine organ? A review of recent data related to cardiovascular complications of endocrine dysfunctions. Clin Exp Hypertens.

[23] Lefebvre AM, Laville M, Vega N (1998). Depot-specific differences in adipose tissue gene expression in lean and obese subjects. Diabetes.

[24] Tchkonia T, Lenburg M, Thomou T (2007). Identification of depot-specific human fat cell progenitors through distinct expression profiles and developmental gene patterns. Am J Physiol Endocrinol Metab.

[25] Ghigliotti G, Barisione C, Garibaldi S (2014). Adipose tissue immune response: novel triggers and consequences for chronic inflammatory conditions. Inflammation.

[26] Drouet M, Dubuquoy L, Desreumaux P, Bertin B (2012). Visceral fat and gut inflammation. Nutrition.

[27] Paeschke A, Erben U, Kredel LI, Kühl AA, Siegmund B (2017). Role of visceral fat in colonic inflammation: from Crohn’s disease to diverticulitis. Curr Opin Gastroenterol.

[28] Gavrilova O, Marcus-Samuels B, Graham D (2000). Surgical implantation of adipose tissue reverses diabetes in lipoatrophic mice. J Clin Invest.

[29] Misra A, Garg A, Abate N, Peshock RM, Stray-Gundersen J, Grundy SM (1997). Relationship of anterior and posterior subcutaneous abdominal fat to insulin sensitivity in nondiabetic men. Obes Res.

[30] Tran TT, Yamamoto Y, Gesta S, Kahn CR (2008). Beneficial effects of subcutaneous fat transplantation on metabolism. Cell Metab.

[31] Moran GW, Dubeau MF, Kaplan GG, Panaccione R, Ghosh S (2013). The increasing weight of Crohn's disease subjects in clinical trials: a hypothesis-generatings time-trend analysis. Inflamm Bowel Dis.

[32] Li Y, Zhu W, Zuo L, Shen B (2016). The role of the mesentery in Crohn's disease: the contributions of nerves, vessels, lymphatics, and fat to the pathogenesis and disease course. Inflamm Bowel Dis.

[33] Desreumaux P, Ernst O, Geboes K (1999). Inflammatory alterations in mesenteric adipose tissue in Crohn's disease. Gastroenterology.

[34] Werkstetter KJ, Ullrich J, Schatz SB, Prell C, Koletzko B, Koletzko S (2012). Lean body mass, physical activity and quality of life in paediatric patients with inflammatory bowel disease and in healthy controls. J Crohns Colitis.

[35] Wiese D, Lashner B, Seidner D (2008). Measurement of nutrition status in Crohn's disease patients receiving infliximab therapy. Nutr Clin Pract.

[36] Subramaniam K, Fallon K, Ruut T (2015). Infliximab reverses inflammatory muscle wasting (sarcopenia) in Crohn's disease. Aliment Pharmacol Ther.

[37] Harries AD, Heatley RV (1983). Nutritional disturbances in Crohn's disease. Postgrad Med J.

