# Supplementary material for: Magnetic resonance imaging-based body composition is associated with nutritional and inflammatory status: a longitudinal study in patients with Crohn's disease
Source: Insights Imaging. 2021 Dec 4;12:178. doi: 10.1186/s13244-021-01121-3 (PMC8643393; doi:10.1186/s13244-021-01121-3)
Supplement: Supplementary file 1 — Additional file 1. Table S1. MRE protocol. Table S2. Kappa coefficients for each intestinal segment in the MRE assessment. Table S3. Correlation between changes in body composition and clinical indicators during treatment (Spearman coefficient or Pearson coefficient). [file 13244_2021_1121_MOESM1_ESM.docx]

**ELECTRONIC SUPPLEMENTARY MATERIAL**

**Table S1.** MRE protocol

|  | T2 WI | T2 WI | T1WI | DWI |
| --- | --- | --- | --- | --- |
| Sequences (GE/ Siemens/United Imaging) | SSFSE/HASTE/SSFSE | FIESTA/TRUFI/BSSFP | LAVA-FLEX/VIBE/DUALECHO | DWI |
| Plane | Coronal, axial | Coronal, axial | Coronal, axial (3D) | Axial |
| Slices thickness (mm) | 4/4/5 | 8/4/5 | 4.2/4.0/3.0 | 4/5/5 |
| TR (ms) | 4000/2000/2500 | 3.2/425.58/4.3 | 3.8/4.5/4.3 | 3000/4700/4000 |
| TE (ms) | 68/80/67.6 | 1.7/1.55/2.1 | 1.7/1.31/1.3 | 80/62/70.7 |

MRE, magnetic resonance enterography; T2WI, T2-weighted image; T1WI, T1-weighted image; DWI, diffusion-weighted image; SSFSE, single-shot fast spin-echo; HASTE, half-Fourier acquisition single-shot turbo spin-echo; FIESTA, fast imaging employing steady-state acquisition; TRUFI, True fast imaging sequence; BSSFP, balance steady-state free precession; LAVA, liver acquisition with volume acceleration; VIBE, volume interpolated body examination; TR, repetition time; TE, echo time; 3D, 3-dimensional

**Table S2.** Kappa coefficients for each intestinal segment in the MRE assessment

| **Segments** | Kappa coefficients | 95% CI |
| --- | --- | --- |
| Jejunum | 0.70 | [0.56, 0.84] |
| Distal ileum | 0.61 | [0.48, 0.74] |
| Terminal ileum | 0.63 | [0.53, 0.73] |
| Ascending colon | 0.81 | [0.74, 0.89] |
| Transverse colon | 0.74 | [0.60, 0.88] |
| Descending colon | 0.71 | [0.58, 0.84] |
| Sigmoid colon | 0.70 | [0.56, 0.84] |
| Rectum | 0.61 | [0.43, 0.80] |

MRE, magnetic resonance enterography; CI, confidence interval.

**Table S3.** Correlation between changes in body composition and clinical indicators during treatment (Spearman coefficient or Pearson coefficient)

|  | ΔBMI | ΔCRP | ΔESR | ΔAlb | ΔHct | ΔTotal MRE score |
| --- | --- | --- | --- | --- | --- | --- |
| ΔSMI | 0.593** | -0.277 | -0.481** | 0.450** | 0.422** | -0.408** |
| ΔSAI | 0.779** | -0.211 | -0.283 | 0.046 | 0.235 | -0.320* |
| ΔVAI | 0.568** | 0.088 | -0.096 | -0.030 | 0.205 | -0.020 |
| ΔVA/TA index | -0.422** | 0.355* | 0.332* | -0.113 | -0.102 | 0.479** |

BMI, body mass index; CRP, C-reactive protein; ESR, Erythrocyte sedimentation rate; Alb, albumin; Hct, haematocrit; SMI, skeletal muscle index; SAI, subcutaneous adipose index; VAI, visceral adipose index

*p<0.05, **p<0.01
